# Investigating the antiproliferative mechanisms of NaHS, a hydrogen sulfide donor, in the SH-SY5Y cell line

**DOI:** 10.1007/s12032-025-02772-8

**Published:** 2025-05-30

**Authors:** Ayşegül Öztürk, Ziad Joha, Neslihan Başgöz, Ahmet Şevki Taşkıran

**Affiliations:** 1 Departments of Therapy and Rehabilitation, Vocational School of Health Services, Sivas Cumhuriyet University, Sivas, Türkiye; 2 Departments of Pharmacology, Faculty of Pharmacy, Sivas Cumhuriyet University, Sivas, Turkey; 3 Department of Physiology, Medicine Faculty, Sivas Cumhuriyet University, Sivas, Türkiye

**Keywords:** NaHS, Hydrogen sulfide, EGFR, Apoptosis, SH-SY5Y

## Abstract

Neuroblastoma, a childhood cancer, arises from neural crest cells within the sympathetic nervous system. This study investigated the anticancer effects of the exogenous hydrogen sulfide (H₂S) donor, sodium hydrogen sulfide (NaHS), on neuroblastoma cells, elucidating how it inhibits cell proliferation. The study was conducted using the SH-SY5Y cell line. The cells were exposed to various concentrations of NaHS (32, 16, 8, 4, and 2 mM) for 24 h. Cell viability was assessed using the XTT assay. ELISA was used to measure levels of the growth signal factors HER-2, EGFR, mTOR, ERK-1, and ERK-2, as well as the apoptosis marker caspase-3. In addition, apoptosis was evaluated using the Muse Annexin V & Dead Cell Assay Kit. NaHS significantly reduced cell proliferation, as demonstrated by the XTT assay (*p *< 0.001). NaHS treatment also decreased levels of HER-2, EGFR, ERK-1, and ERK-2 (*p *< 0.05 to *p *< 0.01), while mTOR levels remained unchanged (*p *> 0.05) and caspase-3 levels increased (*p *< 0.001). Flow cytometry confirmed that NaHS treatment significantly increased the proportion of apoptotic cells (*p *< 0.001). The antiproliferative effect of NaHS against neuroblastoma appears to be mediated through the suppression of certain growth signal factors and the induction of apoptosis. These findings suggest that exogenous hydrogen sulfide donors hold promise as a potential therapeutic strategy for neuroblastoma.

## Introduction

Neuroblastoma is a pediatric malignancy that originates from neural crest cells within the sympathetic nervous system. It is the commonest extracranial solid tumor in children, and its clinical course can vary significantly [1, 2]. Neuroblastoma, which is typically observed in children under the age of five, has an exact cause that is not yet fully understood. The disease is related to genetic mutations, particularly the amplification of the MYCN gene, and various environmental factors [3]. Due to the heterogeneity of neuroblastoma, treatment plans are customized according to prognostic markers and the stage of the disease [4]. In this context, additional research is necessary to develop new treatment methods and effective compounds.

Epidermal growth factor receptor (EGFR/ErbB1/HER1) and other members of the ErbB family, including ErbB2/HER2, ErbB3/HER3, and ErbB4/HER4, are receptor tyrosine kinases that play critical roles in tumor progression across various types of cancer [5]. Abnormal activation of the EGFR is associated with poor outcomes in various malignancies, including non-small cell lung cancer (NSCLC), breast cancer, neck and carcinoma. EGFR activates intracellular signaling pathways through its interaction with ligands, including epidermal growth factor (EGF) and transforming growth factor-alpha (TGF-α). This activation results in dysregulated cellular proliferation [6–9]. EGFR is widely expressed in neuroblastoma cells and primary tumors, where it promotes cell proliferation [5, 10]. Previous studies have demonstrated that EGFR inhibition serves as a promising therapeutic target in neuroblastoma. For this reason, a more comprehensive evaluation of the inhibition of the ErbB family in neuroblastoma is essential [11, 12].

Hydrogen sulfide (H₂S) is a gasotransmitter that plays significant roles in biological systems, both endogenously and exogenously. Endogenous H₂S is a molecule that is naturally produced in our cells and plays a critical role in different biological processes. This molecule possesses neuroprotective, vasodilatory, and antioxidant properties, playing crucial roles in various organ systems, including the nervous and cardiovascular systems. On the other hand, exogenous H₂S, or hydrogen sulfide, is derived from external sources and exerts its effects when introduced into biological systems. Typically, these effects are mediated through chemical donors or compounds that release H₂S [13]. Sodium hydrosulfide (NaHS) is commonly utilized as an exogenous H₂S donor in biological systems. When NaHS is introduced into the body, it dissolves in the aqueous environment, releasing H₂S and thereby mimicking the biological effects of hydrogen sulphide [14]. Studies on the anticancer effects of NaHS suggest that it may modulate cell signaling pathways as a H₂S donor. Nevertheless, the effects of H₂S on cancer cells are bidirectional; in some instances, it may exert anti-carcinogenic effects, while in others, it may have pro-carcinogenic effects [15, 16]. Therefore, elucidating the role and function of H₂S in the progression of neuroblastoma is a significant priority. In this study, we aimed to investigate the anticancer effects of NaHS, an exogenous H₂S donor, on neuroblastoma cancer cells and the mechanisms by which it inhibits cell proliferation in vitro.

## Material method

### Cell line and cell culture

This investigation utilized SHSY5Y (CRL-2266), a human cell line obtained from the American Type Culture Collection (ATCC, USA). SH-SY5Y (neuroblastoma) cells were cultivated in Dulbecco’s Modified Eagle’s Medium (DMEM) supplemented with 10% fetal bovine serum (FBS) and 1% penicillin-streptomycin (Sigma-Aldrich) at 37 °C with 5% CO_2_. NaHS (Sigma, Germany) was dissolved in DMEM.

### Cell viability

Using the XTT test, we assessed the impact of NaHS on the viability of SH-SY5Y cells. The cell line was cultivated at a concentration of 1×10^4^ cells per well and incubated overnight before the addition of NaHS. Subsequently, cells were exposed to various concentrations of NaHS (32, 16, 8, 4, and 2 mM) for 24 hours. As a control, untreated cells were used. After the incubation period, 50 μL of the XTT mixture was added to each well. The cells were shaken after a 4-hour incubation period, and the absorbance was determined at 450 nm using a microplate reader (Thermo Fisher Scientific, Altrincham, United Kingdom). Cell viability was determined by calculating the proportion of live cells compared to untreated cells after three repetitions of each experiment [17, 18]. The IC₅₀ was calculated using GraphPad Prism 8.0 software by fitting a non-linear regression curve to the cell viability data obtained from XTT assays at different concentrations of NaHS.

### The measurement of HER-2, EGFR, mTOR, ERK-1, ERK-2, and Caspase-3 Levels

Elisa commercial kits were used to determine the levels of HER-2 (BT Lab, catalogue #E0224Hu), EGFR (BT Lab, catalogue #E0313Hu), mTOR (BT Lab, catalogue #E3693Hu), ERK-1 (BT Lab, catalogue #E4263Hu), ERK-2 (BT Lab, catalogue #E4264Hu), and caspase-3 (BTLab, catalogue # E4804Hu). Neuroblastoma cells were treated with 19.18 mM NaHS for 24 h. Then, both control cells and NaHS-treated cells were diluted with PBS to a concentration of 1 million cells per milliliter. After three freeze-thaw cycles, supernatants were extracted from the cells. Following the manufacturer's instructions, the levels of HER-2, EGFR, mTOR, ERK-1, ERK-2, and Caspase-3 were measured in the supernatants.

### Protein quantification for ELISA normalization

Total protein concentrations in cell lysates were determined using the Bradford assay kit (SERVA, Germany). The concentrations of target proteins measured by ELISA were then normalized to the total protein content of each sample and expressed as pg/mg of total protein [19, 20].

### Annexin V

Muse Annexin V & Dead Cell Assay Kit (Luminex, Tokyo, Japan) was utilized to assess apoptosis. In this study, SH-SY5Y cells were divided into two experimental conditions: a control group and a NaHS-treated group. After the cells adhered to the culture plate, a NaHS solution was added, and the cells were incubated for 24 h. Apoptosis detection was performed according to the manufacturer's protocol, and the resulting data were analyzed using the Guava® Muse® Cell Analyzer, per the methodologies described in previously published studies [21].

### Statistical analysis

The results of the laboratory were expressed as mean ± standard error. The one-way ANOVA test with a post hoc test was used to analyze the cell viability test data. The independent samples *t*-test was used to examine the data of the HER-2, EGFR, mTOR, ERK-1, ERK-2, caspase-3 level and Annexin V assays. A significance value of *P *< 0.05 was chosen. Data were analyzed, and graphs were generated using GraphPad Prism 8.0 software (USA).

## Results

### SH-SY5Y cell proliferation was reduced by treatment with NaHS

**Fig. 1:**
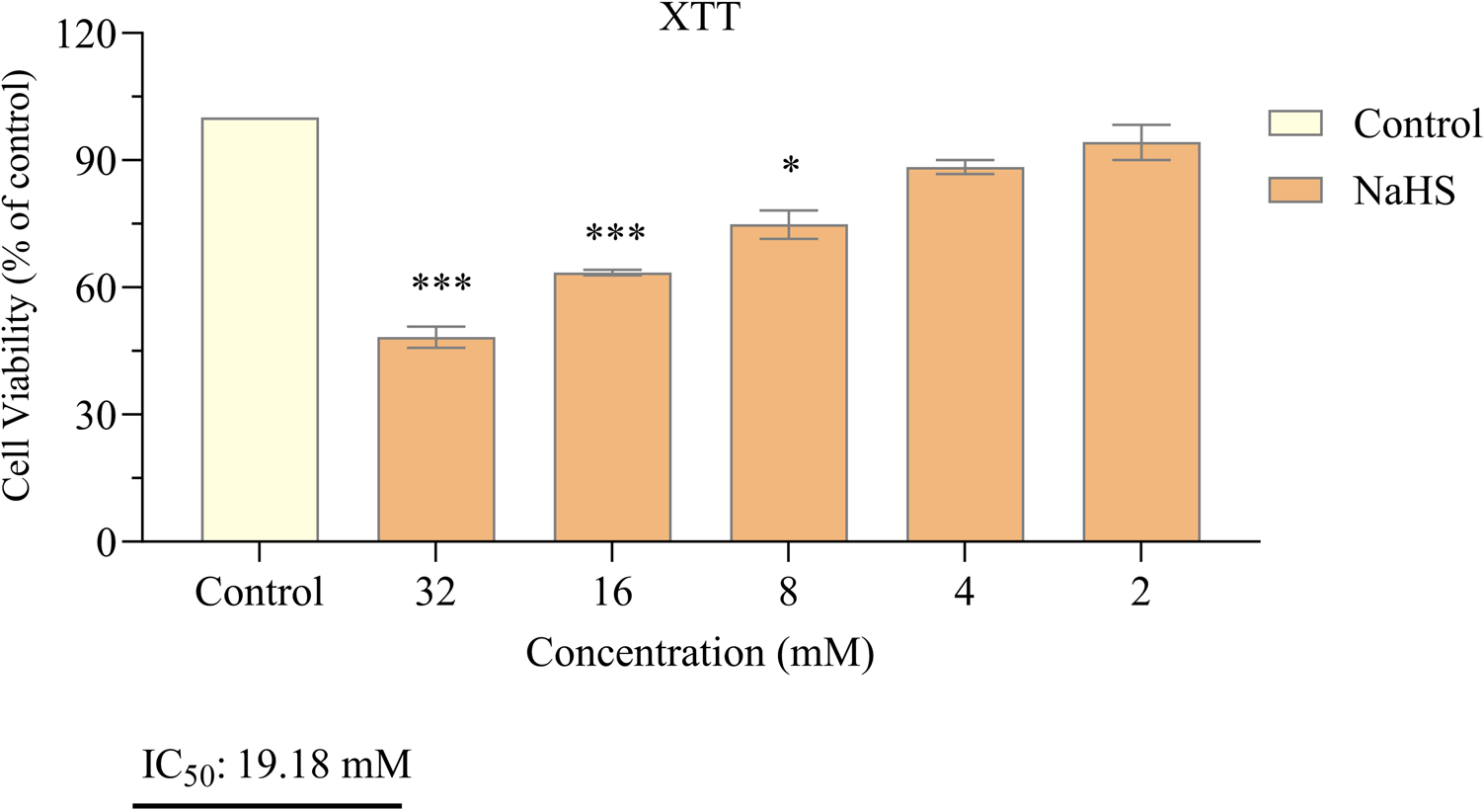
Effect of NaHS administration on SH-SY5Y cell viability. The data are presented as mean ± SEM. Statistical differences between groups were determined using one-way ANOVA followed by Tukey’s post hoc test. *p < 0.05 and ***p < 0.001 were considered statistically significant compared to the control group

**Fig. 2:**
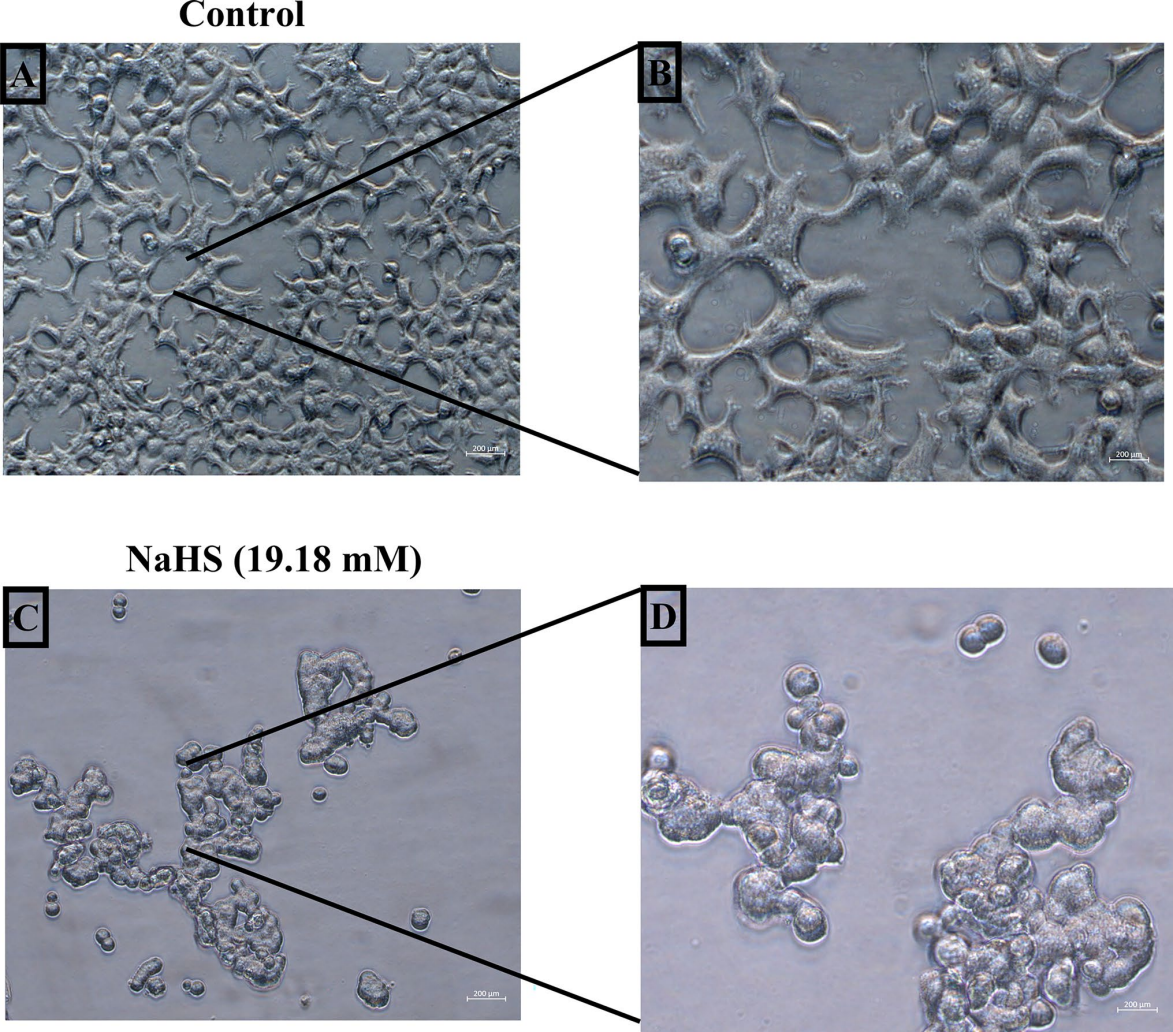
Effects of NaHS (19.18 mM) administration on cell morphology indicating neuronal damage in SH-SY5Y cells. **A** Control group (20X), **B** Control group (40X), **C** NaHS (19.18 mM) (20X), **D** NaHS (19.18 mM) (40X) treated group

The initial step was to investigate the antiproliferative effect of NaHS in neuroblastoma cells. At a concentration of 19.18 mM, NaHS significantly inhibited the proliferation of SH-SY5Y cells compared to the control (*p *< 0.01; Fig. 1). Additionally, light microscopic examinations revealed a decrease in the number of viable cells and an increase in the number of apoptotic cells in SH-SY5Y cells treated with NaHS (19.18 mM), as shown in Fig. 2.

### Effects of NaHS on Growth Factors in SH-SY5Y Cells

**Fig. 3:**
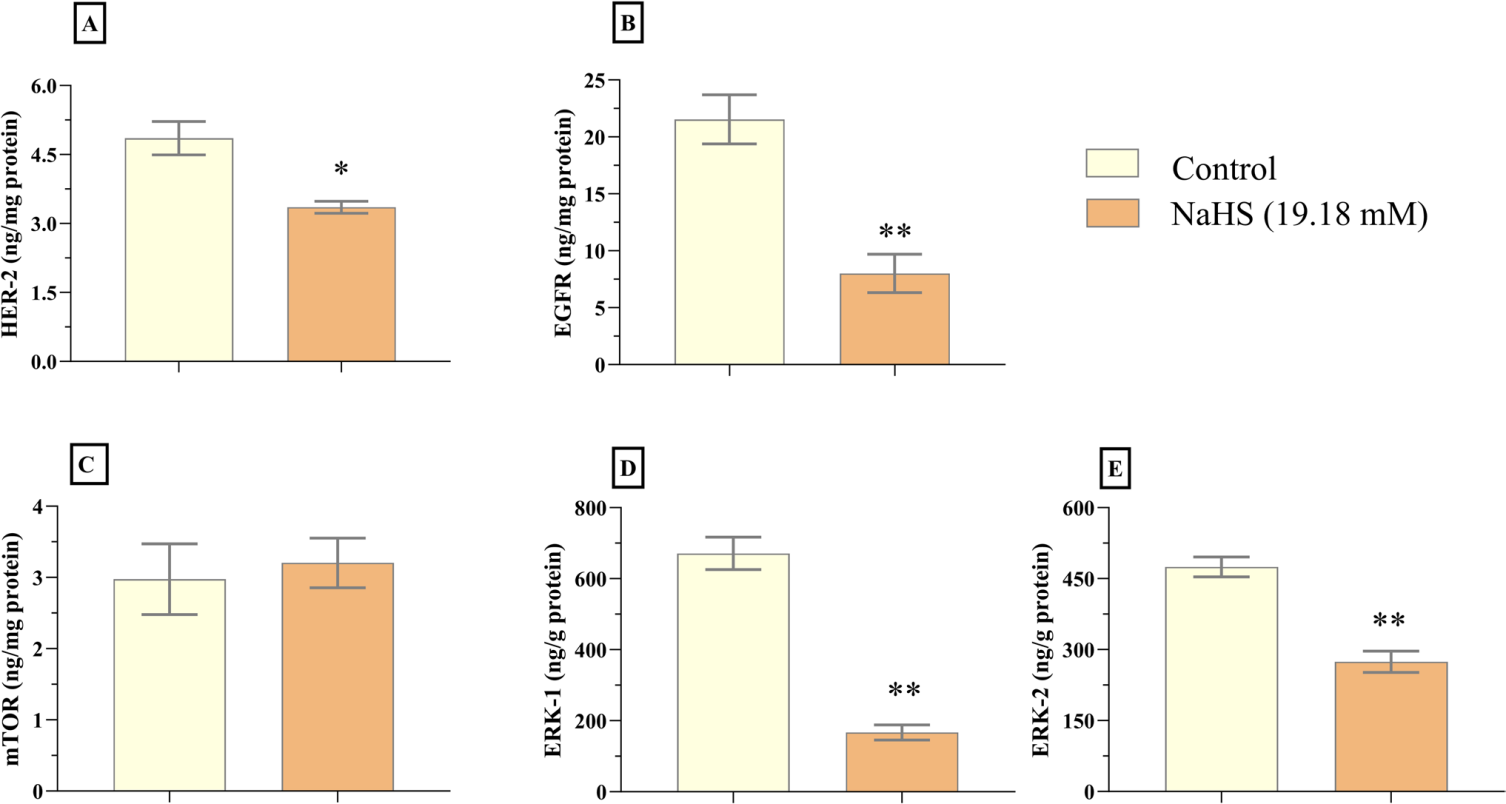
The administration of NaHS (19.18 mM) resulted in a reduction of HER-2, EGFR, ERK-1, and ERK-2 levels in SH-SY5Y cells. The quantities of HER-2, EGFR, ERK-1, and ERK-2 were determined using ELISA kits. The data are given as mean ± SEM. Statistical differences between groups were determined using an independent *t*-test. *p < 0.05, and **p < 0.01 as compared to the control group

ELISA was used to assess the expression of growth factor-related proteins, such as HER-2, EGFR, mTOR, ERK-1, and ERK-2, in SH-SY5Y cells. The administration of NaHS (19.18 mM) for 24 hours resulted in a significant increase in the levels of HER-2 (*p* < 0.05), EGFR (*p *< 0.01), ERK-1 (*p *< 0.01), and ERK-2 (*p *< 0.01) compared to the control group (Figs. 2D, E, 3A, B). However, mTOR levels were not significantly different compared to the control group (*p* > 0.05; Fig. 3C).

### Effects of NaHS on apoptosis in SH-SY5Y cells

**Fig. 4:**
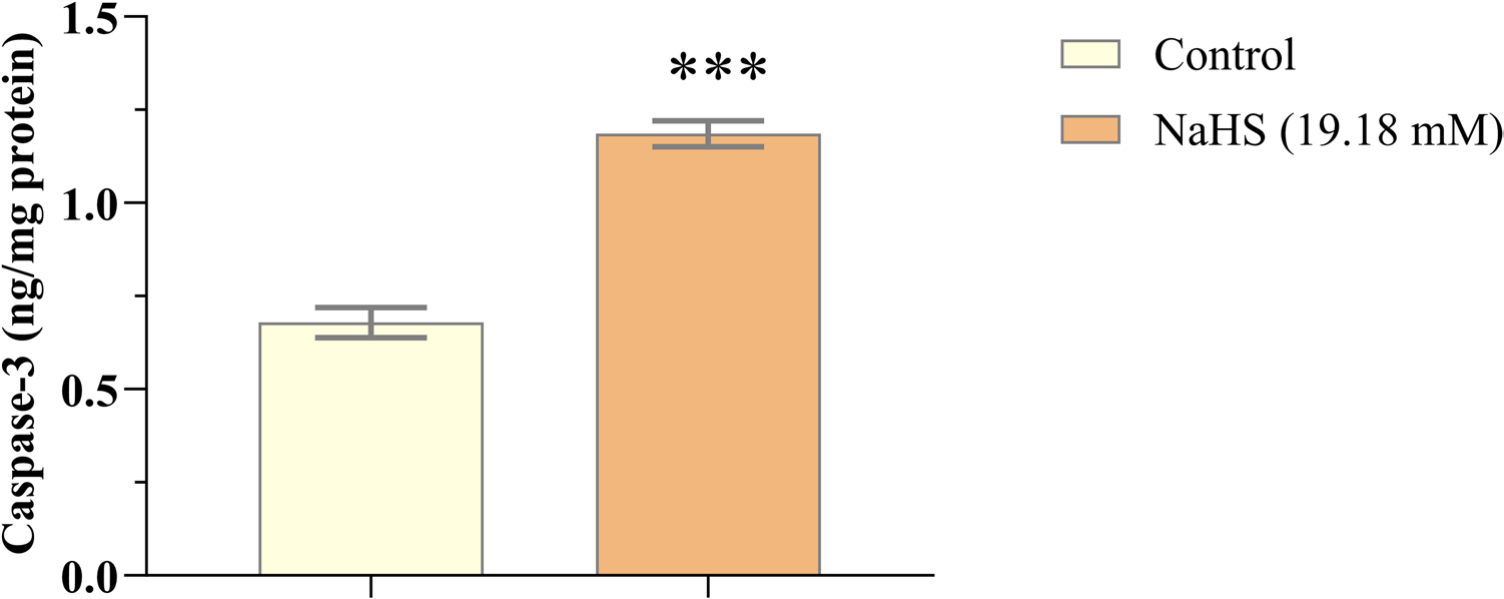
The administration of NaHS (19.18 mM) resulted in an enhancement of Caspase-3 levels in SH-SY5Y cells. The quantity of Caspase-3 was determined using ELISA kits. The data are given as mean ± SEM. Statistical differences between groups were determined using an independent *t*-test. ***p < 0.001 as compared to the control group

**Fig. 5:**
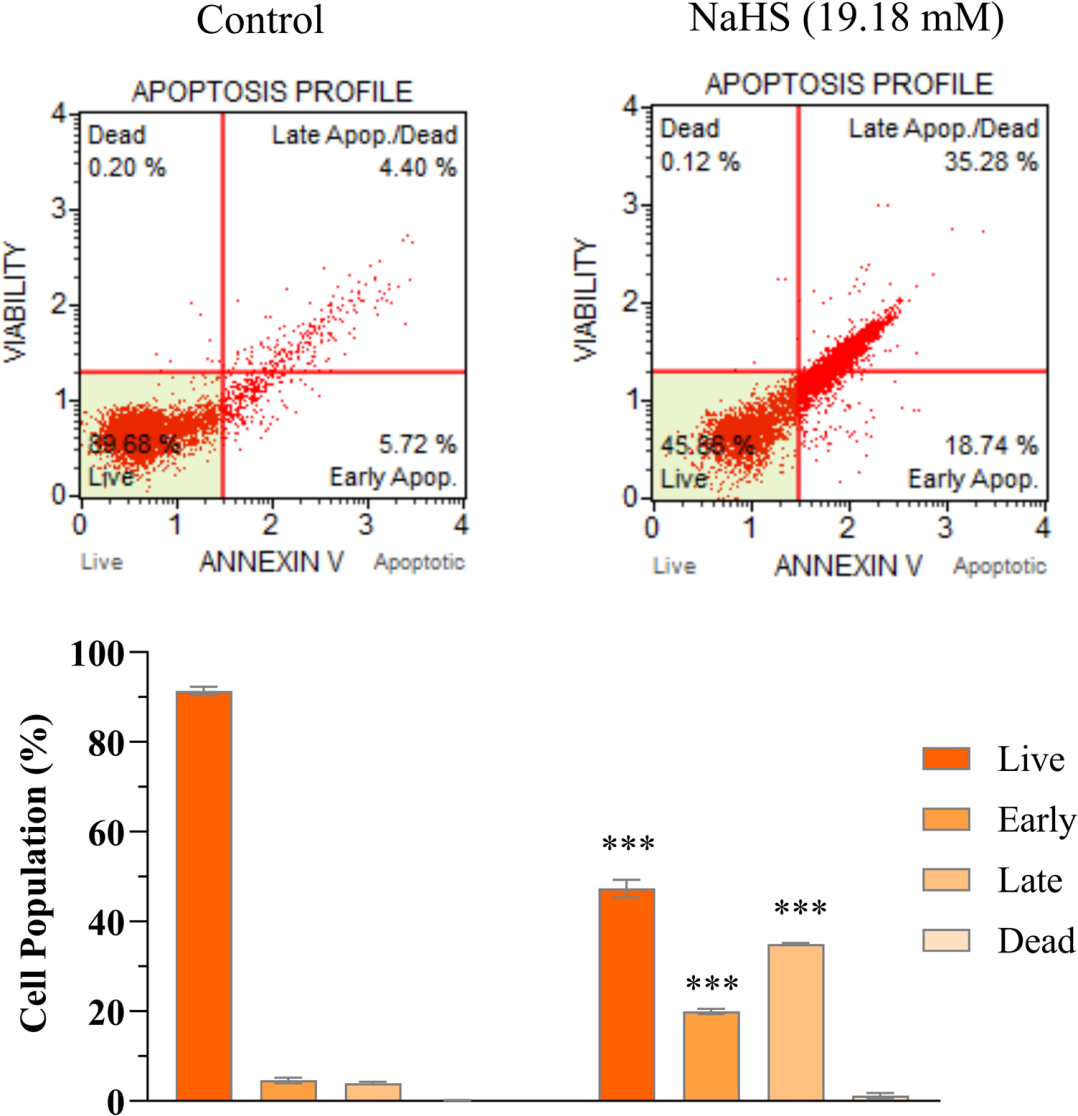
The administration of NaHS (19.18 mM) affects apoptosis as measured by the Annexin V Binding Assay in SH-SY5Y cells. The data are given as mean ± SEM. Statistical differences between groups were determined using an independent *t*-test. ***p < 0.001 as compared to the control group

Caspase-3 levels were measured using an ELISA assay to assess apoptosis in cells. The data obtained indicated that caspase-3 levels were significantly elevated in NaHS-treated SH-SY5Y cells compared to the control group (*p* < 0.05; Fig. 4). In addition, the percentage of apoptotic cells in those treated with a 19.18 mM concentration of NaHS for 24 hours was also evaluated. There was a significant increase in the percentage of apoptotic cells following NaHS treatment, as illustrated in Fig. 5. Flow cytometry analyses confirmed a statistically significant increase in the apoptosis rate in NaHS-treated cells compared to the control group (*p* < 0.001; Fig. 5). The apoptosis rate was significantly elevated in the NaHS-treated cells relative to the control group (*p* < 0.05; Fig. 5).

## Discussion

The relationship between NaHS and H₂S in cancer is complex; the effects of H₂S can both inhibit cell growth and enhance cell proliferation. This bidirectional effect is still under investigation, and no definitive conclusions have been reached regarding the role of H₂S in cancer therapy. Therefore, additional research is required to investigate the potential application of NaHS and hydrogen sulfide in cancer therapy. In this study, we investigated the effects of NaHS used as H₂S donor on cytotoxicity, growth signaling pathways, and programmed cell death (apoptosis) in the SH-SY5Y cell line, a derivative of neuroblastoma, in vitro. The experimental data obtained revealed that NaHS significantly inhibited the proliferation of SH-SY5Y cells in a concentration-dependent manner. The IC50 value was determined to be 19.18 mM following 24 hours of exposure to NaHS. In their study investigating the anticancer activity of NaHS, Zhang et al. found that cell viability in SGC7901 gastric cancer cells decreased in a concentration-dependent manner, which is consistent with our findings [22]. In contrast, Liu et al. reported that the exogenous hydrogen sulfide donor NaHS could induce the proliferation and invasion of cells by increasing the expression levels of MMP-2 and MMP-9 in human bladder cancer EJ cells [23]. Research on colorectal cancer cells have demonstrated that exogenous H_2_S (NaHS) promotes cell proliferation at a concentration of 200 μM in the HCT116 cell line, while concurrently decreasing cell survival at concentrations between 50 and 200 μM in the WiDr cell line [22, 23]. These findings suggest that the effects of different dosages and methods of H_2_S exposure are likely specific to the cell line under investigation. Based on these findings, a bell curve model was proposed to explain the bidirectional effects of H₂S on cancer cells. According to this model, endogenous H₂S or low levels of exogenous H₂S may have a protective effect against cancer cells, while high concentrations or extended exposure to H₂S may result in the death of cancer cells [24]. Lee et al. conducted a study examining the effects of exogenous H₂S donors, specifically NaHS and GYY4137, on both cancerous cell lines (MCF7 and HepG2) and non-cancerous cell lines (MCF10A and WI38). The study revealed that long-term exposure to low concentrations of exogenous H_2_S exhibited selective anticancer effects when compared to a single exposure. Mechanistic analyses have demonstrated that H_2_S disrupts pH regulation by increasing the production of metabolic acids in cancer cells, ultimately leading to cell death. These findings indicate that H_2_S holds significant potential as a selective anticancer agent [25]. In a study, GYY4137, H_2_S donor, was found to inhibit tumor growth by arresting the cell cycle and promoting apoptosis. GYY4137 demonstrated its ability to suppress tumor growth in hepatocellular carcinoma (HCC) cells and in a HepG2 xenograft model by targeting the STAT3 pathway. This effect is mediated through the reduction of phosphorylated STAT3 (p-STAT3) levels, as well as the inhibition of the cell cycle and angiogenesis [26]. However, some studies indicate that NaHS exhibits cytoprotective rather than cytotoxic effects on certain non-malignant cell types under specific conditions. These findings reinforce NAHS's biosafety profile. For example, Denizaltı et al. reported that NaHS enhances cell viability, proliferation, and migration in L929 mouse fibroblast cells. Additionally, it has been demonstrated that NaHS promotes wound healing by reducing oxidative stress under hyperglycemic conditions and may serve as a potential therapeutic agent for skin repair [27]. Similarly, Yu et al. reported that NaHS exhibited protective effects against hemin-induced ferroptosis in BV2 microglial cells. The effect was linked to CBS/H₂S-mediated oxidative stress reduction and increased GPX4, SLC7A11, and GSH levels [28]. These findings suggest that NaHS may not exert cytotoxic effects on certain non-malignant cell types. Supporting this, several studies have demonstrated the cytoprotective and antioxidant effects of NaHS specifically in SH-SY5Y neuroblastoma cells. Whiteman et al. (2005) reported that NaHS (25–125 µM) significantly reduced HOCl-mediated cytotoxicity and oxidative damage in SH-SY5Y cells [29]. Likewise, Whiteman et al. (2004) demonstrated that NaHS (50–250 µM) prevented peroxynitrite-induced protein nitration and cytotoxicity in the same cell line [30]. Koike et al. (2017) further showed that NaHS (200 µM) protected SH-SY5Y cells from methylglyoxal-induced toxicity by activating Keap1/Nrf2 pathways and increasing intracellular glutathione levels [31]. Additionally, Tiong et al. (2010) indicated that NaHS (100–1000 µM) exerted protective effects against 6-hydroxydopamine-induced cell damage through PKCa and PI3K/Akt pathway activation in SH-SY5Y cells [32]. In line with both the literature and our findings, NaHS exhibited protective effects on SH-SY5Y cells at low concentrations, whereas it exerted antiproliferative effects at higher concentrations. A limitation of this study is that the effects of NaHS on healthy cells were not directly investigated. However, previous studies have reported that NaHS may have protective and antioxidant effects in non-malignant cell types. Future studies should include healthy cells to better evaluate the safety and therapeutic potential of NaHS.

To investigate the effect of NaHS on the biological responses of SH-SY5Y cells, we analyzed the expression levels of various proteins involved in growth signaling and apoptosis. EGFR, a member of the receptor tyrosine kinase family, plays a crucial role in various cellular processes and is regarded as a key target for cancer treatment. The activation of EGFR is linked to the proliferation, migration, and invasion of neoplastic cells, and it is found to be overexpressed in several cancer types, including SH-SY5Y neuroblastoma cells [5, 10]. Phosphorylation of the EGFR can activate several signaling pathways, including the ERK1/ERK2, PI3K/AKT/mTOR, and MAPK/MEK/ERK pathways [25, 26]. In a recent investigation, it was observed that NaHS facilitated the phosphorylation of AMP-activated protein kinase (AMPK), inhibited the mTOR signaling pathway, and promoted autophagy in colon epithelial cells [33]. Wu et al. observed that concentrations of 25–50 μM NaHS increased the phosphorylation of EGFR and ERK1/2, as well as angiogenesis, in hepatocarcinoma cell lines (SMMC-7721 and Huh-7), but these phosphorylation and angiogenesis diminished at concentrations of 800–1000 μM NaHS. H₂S had no significant effect on growth, migration, apoptosis, or protein expression levels related to the PTEN/AKT and EGFR/ERK/MMP-2 signaling pathways in normal human liver cells (L02). These findings suggest that H₂S may modulate the migration and growth of hepatocellular carcinoma cells through the EGFR/ERK/MMP-2 signaling pathway [34]. Similar to our findings, NaHS inhibited the EGFR, HER2, ERK1, and ERK2 signaling pathways in the SH-SY5Y cell line. These results indicate that NaHS influences cellular functions in neuroblastoma cells by modulating signaling pathways and that this mechanism contributes to its anticancer activity. mTOR is a kinase that regulates cellular growth, metabolism, and autophagy. Abnormal activation of mTOR is particularly associated with uncontrolled cell proliferation and resistance to apoptosis in cancer [35]. Therefore, mTOR represents a significant therapeutic target for various cancers, including neuroblastoma. However, in our study, we observed that NaHS did not alter mTOR levels. This does not imply that mTOR is not involved in the antiproliferative and pro-apoptotic effects of NaHS. While NaHS decreased upstream signaling molecules such as HER-2, EGFR, and ERK, it may not affect the total level of mTOR. This suggests that NaHS may exert its effects through upstream pathways rather than through direct modulation of mTOR. In addition, the potential changes in the levels of phosphorylated mTOR (p-mTOR), the active form of mTOR, should be considered. Indeed, studies conducted by Wang et al. (2020) and Yu et al. (2009) have revealed that the biological effects and clinicopathological associations of total mTOR and p-mTOR may differ [33, 34]. Therefore, more comprehensive studies are necessary to fully elucidate the role of the mTOR signaling pathway in this process.

Apoptosis is the intrinsic cell death program and serves as a crucial mechanism for maintaining normal developmental processes and tissue homeostasis in multicellular organisms [20, 36]. Apoptosis and the expression levels of caspase-3, a crucial protein associated with apoptosis, were analyzed in detail. Our results indicated that 19.18 mM NaHS significantly enhanced apoptosis. HER2 signaling is primarily mediated by PI3K [37]. The PI3K/AKT pathway plays a crucial role in the metabolism, migration, proliferation, and apoptosis of cancer cells [38, 39]. Wu et al. reported that the BAX/BCL-2 ratio increased when this signaling pathway was inhibited using high concentrations of NaHS [34]. Similarly, Murata et al. investigated the induction of apoptosis in the oral cancer cell line Ca9-22 by H_2_S. H_2_S significantly induced apoptosis in Ca9-22 oral cancer cells. In Ca9-22 cells, H_2_S was found to enhance the expression of a protein known as PHLDA1. This protein subsequently activates caspase-3 during apoptosis, ultimately resulting in cell death [40]. In accordance with previous studies, our findings, based on the data obtained by flow cytometry and ELISA methods, demonstrate that the exogenous H_2_S donor NaHS induces apoptosis in SH-SY5Y neuroblastoma cells. One of the limitations of this study is the absence of caspase-8 and caspase-9 activity measurements, which are crucial for distinguishing between the intrinsic and extrinsic apoptotic pathways. Future research will aim to address this gap.

In conclusion, the suppression of growth signaling pathways by NaHS resulted in a significant increase in caspase-3 levels. These findings suggest that NaHS may operate through a mechanism that enhances apoptosis by inhibiting cell growth in SH-SY5Y cells. This modulation of growth signaling pathways offers a deeper understanding of the effects of NaHS on cancer cells. This study is limited to in vitro cell models, and in vivo validation is necessary. Additionally, further research is needed to investigate the long-term effects and potential toxicities of NaHS. As a next step, we plan to validate our findings in in vivo models and investigate additional apoptotic and proliferative markers. We also aim to explore the potential of NaHS in combination therapies with standard treatments, considering the complex resistance mechanisms in neuroblastoma. Additionally, further research is needed to investigate the long-term effects and potential toxicities of NaHS.

## Data Availability

No datasets were generated or analysed during the current study.
